# Supplementary material for: A comprehensive and scalable database search system for metaproteomics
Source: BMC Genomics. 2016 Aug 16;17:642. doi: 10.1186/s12864-016-2855-3 (PMC4986259; doi:10.1186/s12864-016-2855-3)
Supplement: Additional file 1: — This file contains supplementary figures, methods, and four supplementary tables: Table S1. Data sources used for generation of ComPIL database. Table S2. Adenovirus 5 proteins identified by a ComPIL search of a human HEK293 sample. Table S3. List of proteomes used for generation of the “46 proteomes” database. Table S4. Statistics summary of 3 technical replicates of 5 human fecal samples. (ZIP 1371 kb) [file 12864_2016_2855_MOESM1_ESM.zip › Chatterjee et al 2016 Methods.pdf]

Online Methods

Protein sequence repositories used for construction of databases

Protein records were downloaded from NCBI RefSeq (Release 65), UniProt (Release 2014_06), the NIH Human Microbiome Project reference genomes and HMGI metagenomic sequence repositories (gastrointestinal tract and stool subsections downloaded from http://hmpdacc.org/HMGI/ on 06/14/14 and 02/10/14, respectively), and the Integrated Gene Catalog of human metagenomic sequence data (downloaded on 07/07/14). For the NCBI RefSeq repository, the following subsections of the release were downloaded in protein FASTA format: Viral, Fungi, Mitochondrion, Plasmid, Protozoa, Archaea, Bacteria. For the UniProt database, the human proteome and the complete proteomes for subsections Archaea, Bacteria, and Viruses were downloaded and incorporated. The HMGI metagenomic FASTA records were filtered to remove records containing the amino acid 'X' (appearing in ~5% of records from this repository). All other protein sequences were incorporated without processing or alteration, which resulted in a total of 82,817,736 protein sequences. The protein FASTA file is available for download at https://hpccloud.scripps.edu/index.php/s/xnVZo6THCi7jjrk (MD5 checksum 1104ceac3f1154cb4d70f1c0b3677d08).

Hardware and software details

Database generation was performed on a Dell R420 Linux server with an Intel Xeon E5-2450 16-core CPU with 96GB RAM and two 4TB hard disk drives running CentOS 6.5, Python 2.7.5, Python 3.4.3, Java 1.7.0_55 (OpenJDK), GNU Parallel 2014_0622, GNU Sort 8.22, and MongoDB 3.0.6. Databases were either stored locally on this same Dell server or on an 8-node Aeon Computing Eclipse Microcloud Server System, with each Microcloud node outfitted with an Intel Xeon E5-1650 v2 6-core CPU with 64GB RAM and a 512GB Samsung 840 Pro solid state drive. Microcloud nodes were running CentOS 6.5 with MongoDB 3.0.6, with each node acting as a separate shard in an 8-node sharded database. Proteomic searches were performed using a ~5000-core Linux cluster located in the TSRI high-performance computing core facility. A single 'mongos' query router process was started on one node per network switch in the computing cluster to connect to database shards. Software and source code is publicly available at https://bitbucket.org/sulab/metaproteomics

Generation of ComPIL databases

FASTA protein records from all sequence repositories were concatenated together in a single file containing 82,817,736 protein sequences. Individual protein sequences were reversed to generate decoy proteins, and forward and reversed protein sequences together resulted in one ~60GB FASTA file with 165,635,472 protein sequences. All protein entries were numbered with a unique integer ID (1 to 165,635,472) and imported into MongoDB (as 'ProtDB') using the schema shown in Figure S1. Tryptic peptides (*i.e.*, cleavage after lysine and arginine residues except before proline) were generated *in silico* using Blazmass for each protein sequence with the constraint that all peptides be fully tryptic (*i.e.,* have two tryptic ends) and a maximum of three internal missed cleavage sites. The format of the peptide output was a tab-delimited file with fields: (1) peptide mass (rounded to 3 decimal places, multiplied by 1000, and stored as an integer), (2) tryptic peptide sequence, (3) 3 residues N-terminal to the peptide in its parent protein sequence, (4) 3 residues C-terminal to the peptide in its parent protein sequence, (5) the parent protein integer ID, and (6) the start position of the peptide in its parent protein sequence. Using GNU sort, this large peptide file was sorted either by peptide mass or by peptide sequence to group peptides by mass or sequence, respectively. Groups of peptides with the same mass or the same sequence were then combined together into JSON objects. After importing into MongoDB, the peptide mass-grouped JSON objects comprise 'MassDB' and the

peptide sequence-grouped JSON objects comprise 'SeqDB.' A schematic of this database generation workflow is shown in **Figure 1C**.

Generation of human and *Bacteroides fragilis* subset databases

Generation of the human and *Bacteroides fragilis* protein and peptide databases was performed as described above, except with an initial input FASTA file of all proteins from organism names beginning with the string 'Homo sapiens' or 'Bacteroides fragilis'. These subset FASTA files were generated using MongoDB 2.6.3, Python 3.3.2, and PyMongo 2.7.1 with one of the following ComPIL ProtDB database queries: collection.find({"o":{"$regex":"Homo sapiens.*"}}) or collection.find({"o":{'$regex':"Bacteroides fragilis.*"}}).

Generation of "46 proteomes" subset database

Generation of the "46 proteomes" protein and peptide databases was performed with the following ComPIL ProtDB query structure: collection.find({"o":{"$in":["organism_name_1", "organism_name_2", …]}}) where the organism names used are listed in **Table S3** ("Homo sapiens" was added to this list). The organisms used for the "46 proteomes" database are a subset of a list of 51 organisms previously used in a human gut microbiome metaproteomic analysis.

Mass spectrometry proteomic searches

Precursor ion and fragmentation data were extracted from Xcalibur raw files using RawXtract 1.9.9.2 (http://fields.scripps.edu/downloads.php). The resulting MS2 files were searched using Blazmass integrating ComPIL/MongoDB connectivity and traditional cross-correlation scoring. MS/MS scans were searched using a precursor mass tolerance of 10 ppm for microbiome searches or 30 ppm and a fragment ion mass tolerance of 50 ppm. A static modification was specified on cysteine (+57.02146) and a differential modification was specified on methionine (+15.9949). MS2 files were each split into 25-50 chunks in order to parallelize database searches using the Linux cluster located in the TSRI High Performance Computing (HPC) core. Chunks were recombined after searches were complete. Blazmass source code and installation instructions are available at https://github.com/sandipchatterjee/blazmass_compil

Filtering and false discovery rate calculation

The top-scoring peptide match of each scan in the Blazmass SQT output was queried against SeqDB and ProtDB to generate a subset FASTA file consisting of all peptide sequences' parent proteins, including the false reversed (decoy) protein sequences. This FASTA file and the SQT output were used as input for filtering using DTASelect 2.1.3 (http://fields.scripps.edu/downloads.php), at a protein false positive rate of 1%, requiring 2 peptides per protein. The following parameters were used: "--quiet --brief --trypstat --modstat -y 2 -DM 10 --extra --dm --pfp 0.01 -p 2".

Protein cluster generation

All forward protein sequences were clustered using CD-Hit using a sequence identity threshold of 70% and a length difference cutoff of 50%, with fast cluster mode disabled. Parameters used: cd-hit -i $FASTA -o $FASTAOUT -c 0.7 -n 5 -d 0 -M 98000 -T 16 -g 1 -s 0.5. This resulted in 17,342,571 protein clusters. In order to analyze proteomic samples, peptides identified in a sample are built into protein loci (similarly to DTASelect) and the protein loci are mapped into protein clusters. Using this technique, each sample is quantified using a common set of protein clusters.

Functional annotation of proteins and protein clusters

The unique set of 53,150,335 protein sequences (unique sequences within the 82,817,736 'forward' protein sequences) were queried against the PfamA, TIGRFAM, Panther, SMART, PrositePatterns, PRINTS, Gene3d, and Coils databases using InterProScan v5 (version 5.8-49.0) using default parameters. InterPro, Gene Ontology, and Pathway annotation were enabled. A copy of the pre-calculated match lookup service was downloaded and queried locally. Queries were divided into chunks of 5,000 - 20,000 sequences each and searched using the HPC core, taking approximately 4-6 hours per chunk, with the output stored as a tab-separated file. The resulting output files were parsed into JSON objects and imported into a MongoDB database. A total of 39,800,965 protein sequences (75%) had one or more match against any of the InterPro member databases. A protein cluster was annotated with all GO terms associated with any domain for all possible proteins within that cluster, while removing any GO terms that were parents ("is a" or "part of" relationships) of other GO terms in that protein cluster.

Identification of Human Adenovirus proteins
The HEK293 SQT output was filtered and the resulting peptides joined into protein loci using DTASelect 2.1.3 with a minimum requirement of 2 peptides per protein and a maximum peptide FDR of 0.1%. The taxonomy ID of each protein was looked up using a Python script that parses each FASTA defline for an organism name and matches the organism's scientific name to its taxonomy ID. Proteins without taxonomy information were skipped. For each protein locus, the lowest common ancestor was determined using a Python script. Taxonomy information was parsed from flat-files provided by the NCBI: ftp://ftp.ncbi.nih.gov/pub/taxonomy/taxdump.tar.gz

Human proteome isolation
HEK293 cells were grown under 5% CO_2 at 37 °C in EMEM minimum essential medium (Quality Biological Catalog # 25030-081) supplemented with 2 mM L-glutamine, 1X MEM non-essential amino acids, 1X penicillin-streptomycin, 1 mM sodium pyruvate, and 10% (v/v) Heat Inactivated Fetal Bovine Serum, all from Gibco/Life Technologies. After cultures reached approximately 80% confluence, cells were gently scraped into DPBS and pelleted by centrifugation. Each cell pellet was rinsed twice with cold DPBS and frozen at -80 °C after removing all buffer. Cells pellets were later thawed on ice and resuspended in a solution of DPBS and Roche cOmplete protease inhibitor cocktail (Roche Catalog # 04693159001). The resuspended cell pellet was subjected to sonication in a Qsonica Q700 sonicator with Cup Horn attachment (water bath held at 4 °C) using a program of 1 sec on / 1 sec off at 50% amplitude for 3 x 1-min cycles.

***Bacteroides fragilis* proteome isolation**
Bacteroides fragilis (ATCC Catalog # 25285) cultures were grown for 24 hours at 37 °C under anaerobic conditions in Brucella broth (Anaerobe Systems Catalog # AS-105). Cells were harvested by centrifugation at 4 °C followed by removal of the supernatant growth medium and freezing at -80 °C. Cells pellets were later thawed on ice and resuspended in a solution of DPBS and Roche cOmplete protease inhibitor cocktail (Roche Catalog # 04693159001). The resuspended cell pellet was subjected to sonication in a Qsonica Q700 sonicator with Cup Horn attachment (water bath held at 4 °C) using a program of 1 sec on / 1 sec off at 50% amplitude for 3 x 1-min cycles.

Human stool proteome isolation
Human stool samples were collected by healthy human donors and frozen immediately on dry ice. Samples were later thawed, divided into 150-200 mg aliquots, and refrozen at -20 °C. Aliquots were resuspended in 500 µL of a solution of Roche cOmplete protease inhibitor (Roche Catalog # 04693159001) in DPBS. Samples were then subjected to sonication in a Qsonica Q700 sonicator with Cup Horn attachment (water bath held at 4 °C) for 10 minutes at 50%

amplitude. The lysate was then clarified by centrifugation at 21,000*g* for 20 minutes at 4 °C, and the soluble supernatant was transferred to a new tube.

Proteome sample preparation for mass spectrometry

Protein concentrations of all cell lysates were measured against a BSA standard curve using the Pierce BCA Protein Assay Kit. 25μg of each protein sample was diluted to a final volume of 100 μL in DPBS. 30 μL of 6.1N trichloroacetic acid (Sigma-Aldrich Catalog # T0699) was added to each 100 μL sample, samples were mixed thoroughly, and placed at 4 °C overnight. Precipitate was collected the next morning by centrifugation at 21,000*g* for 20 minutes at 4 °C. Protein pellets were washed twice with cold acetone (JT Baker Catalog # 9002-02) and allowed to air dry at room temperature for several hours. Dry protein pellets were solubilized in 60 μL of a solution of 8 M Urea in 100 mM Tris-HCl (pH 8.5). Cystine disulfide bonds were reduced by addition of *tris*(2-carboxyethyl)phosphine (TCEP) to each sample to a final concentration of 5 mM and incubation at room temperature for 20 min. Cysteine residues were subsequently alkylated by addition of 2-chloroacetamide to a final concentration of 50 mM for 15 min at room temperature. Samples were diluted to 240 μL volume with 100 mM Tris-HCl (pH 8.5), supplemented with 1 mM $CaCl_2$ and 1 μg trypsin (Promega Catalog # V5111), and samples were incubated at 37 °C overnight. Formic acid (Fisher Catalog # A117-50) was added to each tube (5%, v/v) the following morning, and samples were centrifuged for 15 minutes at 21,000*g*. The supernatant was collected and frozen at -20 °C.

LC column preparation and sample loading

MudPIT columns were prepared in-house as previously described [23]. Briefly, strong cation exchange (SCX) and reverse-phase C18 dry resins were sequentially pressure-loaded into a 250-μm (internal diameter) fused silica capillary (Agilent Catalog # 160-2250-10) such that 2 centimeters of each resin were adjacent to each other. 12 centimeters of C18 resin were pressure-loaded into a 100-μm (internal diameter) fused silica capillary that had been pulled to a fine point using a pipette puller instrument (Sutter Instrument Company, Model P-2000). Acidified peptide samples were pressure-loaded on to the biphasic column, and the biphasic column was connected to the reverse-phase tip using a specialized union connector (IDEX Catalog # P-720-01).

Mass spectrometry data collection

Tandem mass spectrometry data for each sample was collected on a Thermo Scientific Orbitrap Velos Pro coupled to an Agilent 1200 series HPLC quaternary pump. Peptides were eluted off the biphasic MudPIT column and separated over 10 chromatography steps, each of which consisted of a 3-minute "salt bump" of between 10% and 100% solvent C, followed by a 100-minute linear gradient of 0% to 35% solvent B (solvent A: 95% H_2O, 5% acetonitrile, 0.1% formic acid; solvent B: 20% H_2O, 80% acetonitrile, 0.1% formic acid; solvent C: 500 mM ammonium acetate, 95% H_2O, 5% acetonitrile, 0.1% formic acid). An eleventh and final chromatography step consisted of a 3-minute 90% solvent C/10% solvent B "salt bump" followed by a 100-minute linear gradient of 0% to 35% solvent B. Precursor ions were recorded by scanning in the range of m/z 400.00-2000.00 with the FTMS analyzer and a resolution of 60,000. The top 10 peaks were selected for fragmentation using HCD with normalized collision energy set to 35.0. Fragmentation data were also collected in the FTMS analyzer, but with a resolution of 7500. Dynamic exclusion was enabled with exclusion duration set to 20.0 seconds.

Microbiome DNA preparation and gene

Microbial DNA was isolated from fecal samples with a fecal DNA MiniPrep extraction kit (Zymo Research). Isolated DNA was subjected to standard PCR-based cloning to amplify segments of the *Blautia wexlerae* WP_025578033 containing a DUF PF09861 superfamily sequence.

Amplification was performed with 2 sets of primer pairs, including forward 5'-ggctgcttatctggaaagtctggctcatcc-3' and reverse 3'-ccagtacagcatcacagcagaagaaagg-5' primers as well as forward 5'-gacaagagaacctatgtacactgggcagag-3' and reverse 3'-cccattcctcttgcatagcccggttcc-5' that result in 647 and 461 base pair products, respectively (**Figure S7A**). DNA sequencing confirmed both products aligned with 100% agreement to the predicted gene sequence.

Protein preparation, and purification

The full-length *B. wexlerae* DUF PF09861 clone (residues 1-495) was generated using standard PCR-based cloning with forward primer 5'-aagctagcaaatttgattttgagtatggacagg-3' and reverse primer 3'-aactcgagacagcaacatggatgacgatgtcc-5' engineered to contain NheI and XhoI restriction enzyme cut sites (underlined), respectively, for insertion into a pET23b vector (Novagen). The resulting clone with a C-terminal His6-tag fusion was verified via double-stranded plasmid sequencing. *B. wexlerae* DUF is overexpressed from *E. coli* BL21DE3pLysS (Strategene) and cells were grown in 2xYT media supplemented with 200 mg/mL ampicillin and 50 mg/mL chloramphenicol at 37 °C to an OD600nm of 0.75. Flasks were then transferred to 18 °C and protein expression was induced with 0.2 mM IPTG overnight. Cells were harvested and resuspended in ice cold 100 mM Tris, pH 8.0, 100 mM NaCl (buffer A) and subjected to 3 cycles of lysis by microfluidization (Microfluidics). The cell lysate was clarified by centrifugation at 45,000x*g* for 30 minutes at 4 °C and soluble fractions were loaded onto a 1 mL HisTrap HP Ni-NTA affinity column (GE Amersham) pre-equilibrated with buffer A and eluted with buffer A containing 250 mM Imidazole. The eluted protein was immediately diluted 5-fold with buffer B (20 mM Tris, pH 8.0) and purified by cation-exchange chromatography (HiTrap SP HP, GE Amersham) with a 30-column volume gradient to 75% of buffer B containing 1 M NaCl. Fractions corresponding to DUF were pooled and concentrated to approximately 10 mg/mL using Millipore Ultrafree-15 devices with a MWCO of 3,500 Da and immediately stored at -70 °C. Pure DUF yields are approximately 5 mg/L of culture with >95% purity, as assessed by SDS-PAGE (Figure S7).

Large database search with SearchGUI

SearchGUI-2.8.0 was tested on a Dell R420 Linux server with an Intel Xeon E5-2450 16-core CPU with 96GB RAM and two 4TB hard disk drives running CentOS 6.5. SearchGUI includes the following search engines: Andromeda, Tide, MS Amanda, X!Tandem, MyriMatch, MS-GF+, OMSSA, and Comet. Andromeda was not tested as it is not available for Linux. Tide and MS Amanda were not tested due to operating system incompatibilities. MS-GF+, X!Tandem, OMSSA, MyriMatch, and Comet were tested.

The original FASTA file used to generate the ComPIL database was reformatted to SearchGUI's specifications, and a small section of a mass spectrometry file with a limited subset of 100 scans (out of the total 238,210 scans) was used for testing. Attempts to search the ComPIL database using MS-GF+, X!Tandem, MyriMatch and OMSSA were unsuccessful due to insufficient RAM. Only Comet was successfully able to search this small MS file.

The Comet search against 100 scans required approximately 3 minutes per scan per core (32 cores, 5 h 27 min total) in comparison to approximately 1 second per scan per core (32 cores, 2 min total) for Blazmass-ComPIL. We employ a standard 10-step MudPIT MS/MS data collection that results in approximately 200,000 scans per sample. Therefore, with an average 50x parallelization, Comet would take at least 8 days to search a single sample. Conversely, ComPIL ability can complete the search in 1-2 hours. As such, these computing limitations prevented us from analyzing a full set of MS/MS data scans from one sample with Comet. A

machine with >96GB of RAM and/or a significant investment in external computational resources would likely be required to accurately evaluate the speed or accuracy of other search engines with the ComPIL database. Notwithstanding, the Comet to Blazmass comparison performed on the 100 scans subset resulted in a filtered PSM agreement of 100%, as defined by both search engines matching the same peptide without post-translational modifications as the top match. Filtered PSMs were compared using the Blazmass-defined filtered dataset, which was based on the entire sample being searched.
