# Supplementary material for: A comprehensive and scalable database search system for metaproteomics
Source: BMC Genomics. 2016 Aug 16;17:642. doi: 10.1186/s12864-016-2855-3 (PMC4986259; doi:10.1186/s12864-016-2855-3)
Supplement: Additional file 1: — This file contains supplementary figures, methods, and four supplementary tables: Table S1. Data sources used for generation of ComPIL database. Table S2. Adenovirus 5 proteins identified by a ComPIL search of a human HEK293 sample. Table S3. List of proteomes used for generation of the “46 proteomes” database. Table S4. Statistics summary of 3 technical replicates of 5 human fecal samples. (ZIP 1371 kb) [file 12864_2016_2855_MOESM1_ESM.zip › Chatterjee et al 2016 Supp Figures Revision.pdf]

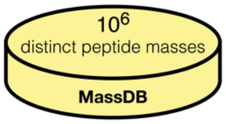


```
{
   "_id" : (mass value rounded to 3 decimal places and
           multiplied by 1000, integer),
   "s" : (array of peptide sequences with this mass, string)
}
```

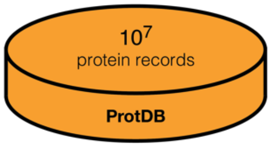


```
{
   "_id": (PROT_ID, unique protein identifier, integer),
   "d": (FASTA defline, string),
   "s": (complete protein sequence, string),
   "r": (source repository for protein record, string),
   "o": (source organism [Genus] [species] [strain], string) — OPTIONAL,
   "gb": (array of GO Biological Process terms) — OPTIONAL,
   "gc": (array of GO Cellular Component terms) — OPTIONAL,
   "gm": (array of GO Molecular Function terms) — OPTIONAL
}
```

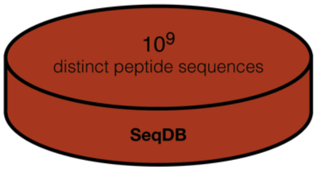


```
{
   "_id" : (unique peptide sequence, string),
   "p" : (PARENTS, array of JSON objects -- one object per protein)
     [ {
        "i": (PROT_ID, unique protein identifier, integer),
        "r": (RESRIGHT, 3 residues to right of peptide end position, string),
        "l": (RESLEFT, 3 residues to left of peptide start position, string),
        "o": (OFFSET, peptide start position in protein sequence, integer),
        "d": (DECOY, whether or not protein sequence is a decoy, boolean)
            – OPTIONAL
     },
     {
        "i": (PROT_ID, unique protein identifier, integer),
        "r": (RESRIGHT, 3 residues to right of peptide end position, string),
        "l": (RESLEFT, 3 residues to left of peptide start position, string),
        "o": (OFFSET, peptide start position in protein sequence, integer)
     },
     ...
     ]
}
```

Figure S1 – Organization (schema) for MassDB, ProtDB, and SeqDB. MassDB contains an array of tryptic peptide sequences associated with distinct peptide masses. ProtDB contains protein sequences with a unique protein record identifier, grouped with organism and gene ontology annotation for each protein (when available). SeqDB contains distinct peptide sequences associated with a list of parent proteins for each peptide.

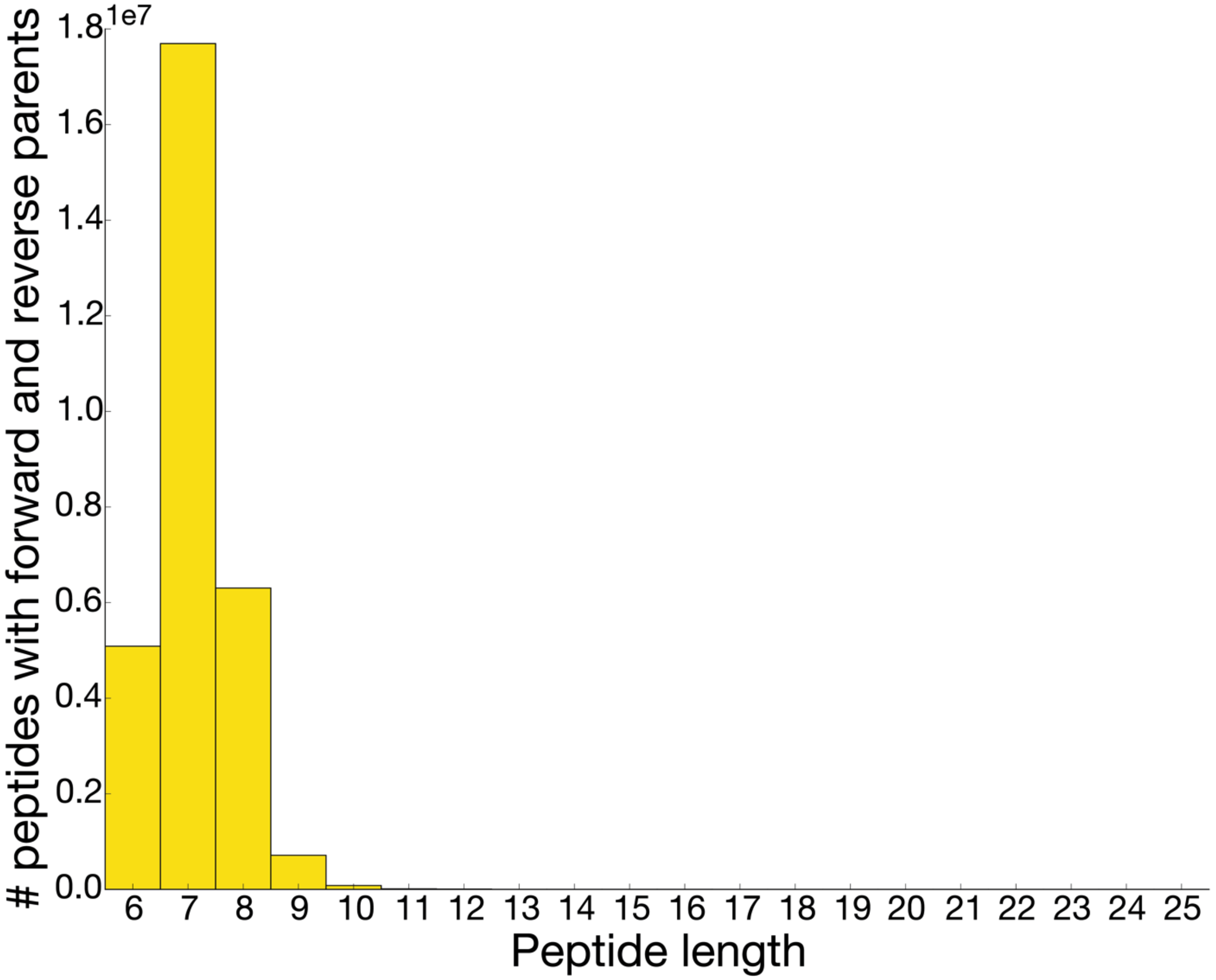

Figure S2 – Peptide sequence overlap for peptides from real ('forward') and decoy ('reverse') proteins, by peptide length. Only 0.7% of all ComPIL peptide sequences have both a real and decoy parent protein, and the majority of these peptides are short peptides, which are not commonly observed in filtered shotgun proteomics data.

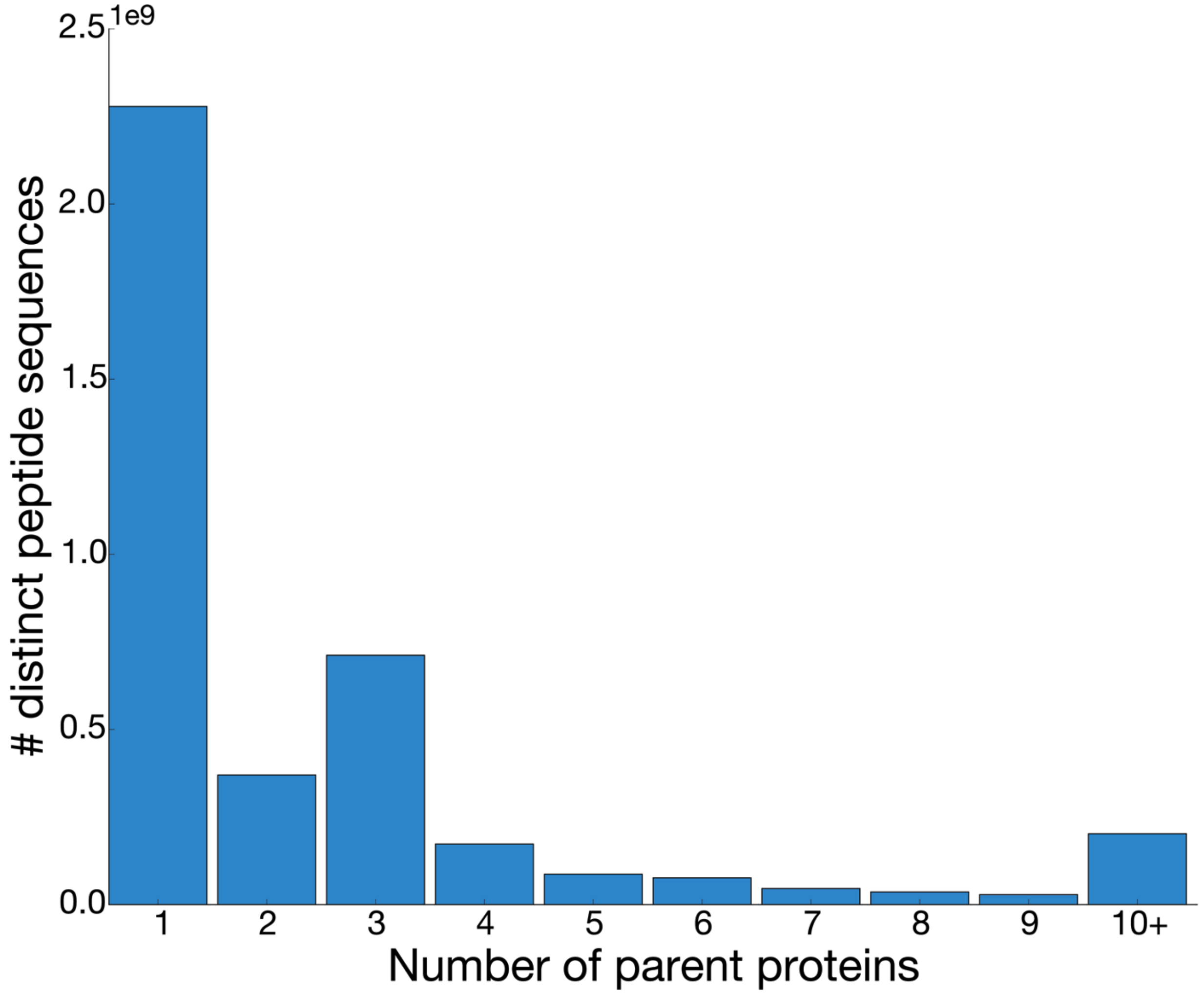

Figure S3 – Parent proteins for distinct ComPIL tryptic peptide sequences. 57% of tryptic peptide sequences appear in only one ComPIL protein, and 84% of peptide sequences appear in 3 or fewer ComPIL proteins.

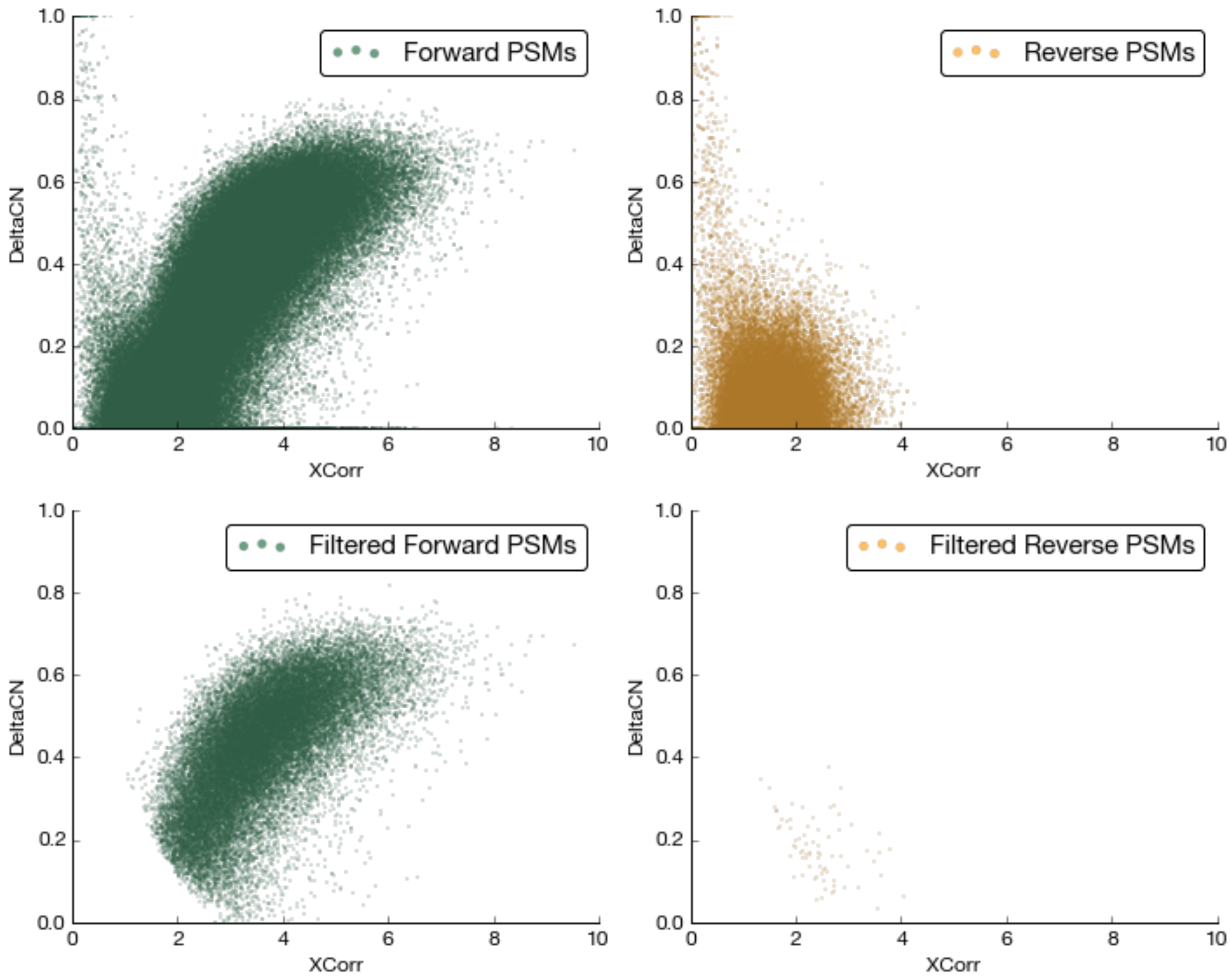

Figure S4 – DeltaCN vs. XCorr for the HumanDB search of HEK293 tandem mass spectra. HumanDB searches of HEK293 cells. Forward PSMs (top left) and reverse (decoy) PSMs (top right) are shown before filtering. Filtered forward PSMs (bottom left) and filtered reverse PSMs (bottom right) are shown after filtering at 1% FDR with DTASelect2.

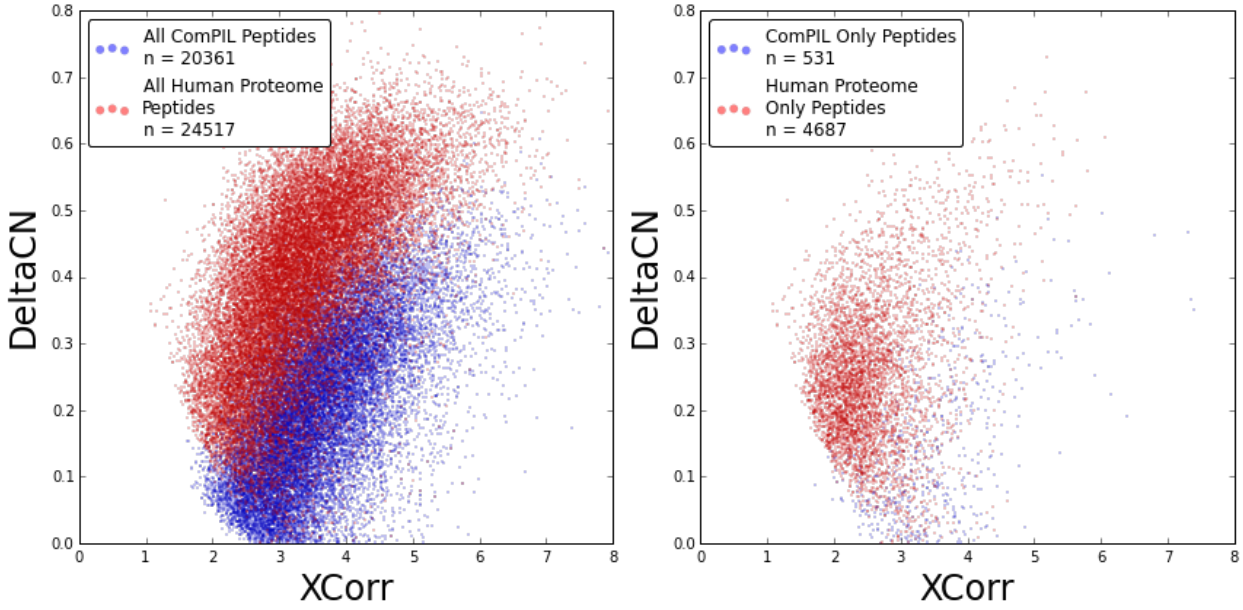

Figure S5 - DeltaCN vs. XCorr for the ComPIL & humanDB search of HEK293 sample. All peptides found in the ComPIL and humanDB search are shown (left) as well as the peptides that are found in only the ComPIL search or only the humanDB search (right).

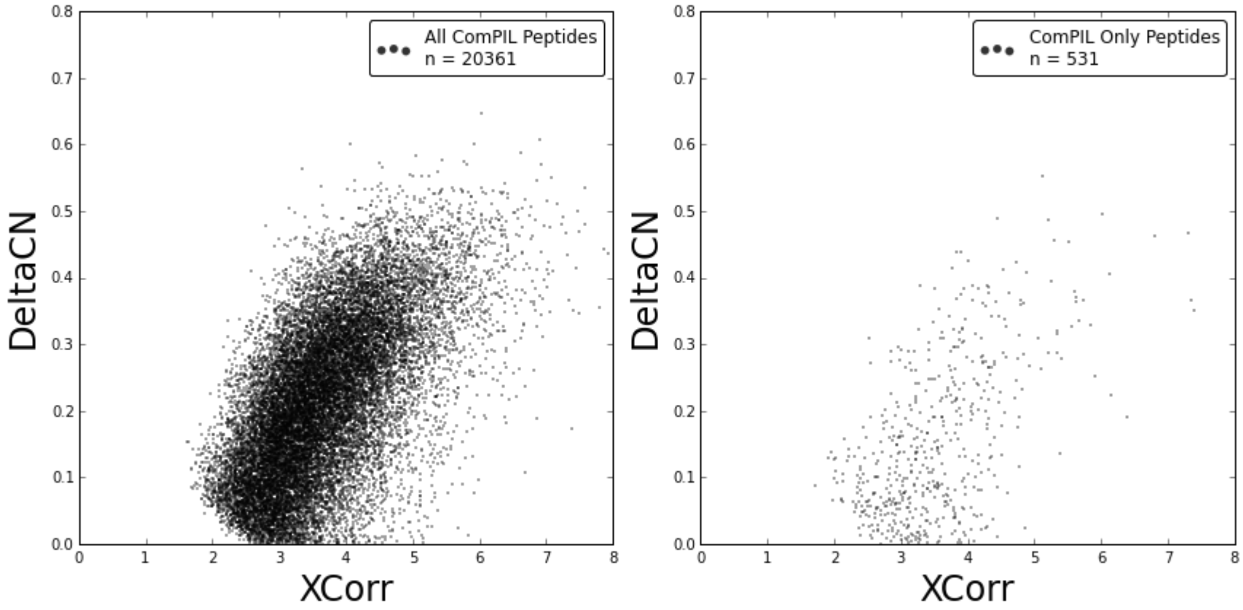

Figure S6 - DeltaCN vs. XCorr for the ComPIL vs. humanDB search of HEK293 sample. All peptides found in the ComPIL search are shown (left), along with the peptides that are found in the ComPIL search only (not found in the humanDB search) (right).

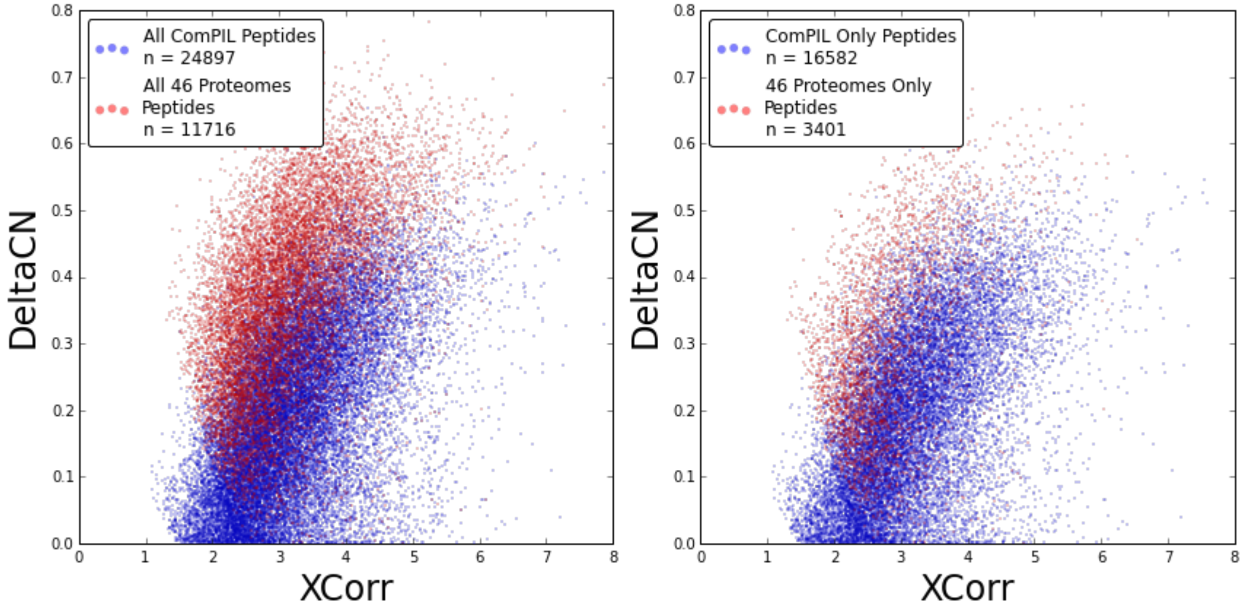

Figure S7 - DeltaCN vs. XCorr for the ComPIL & 46 proteomes search of the H1_1 fecal microbiome sample. All peptides found in the ComPIL and 46 proteomes search are shown (left) as well as the peptides that are found in only the ComPIL search or only the 46 proteomes search (right).

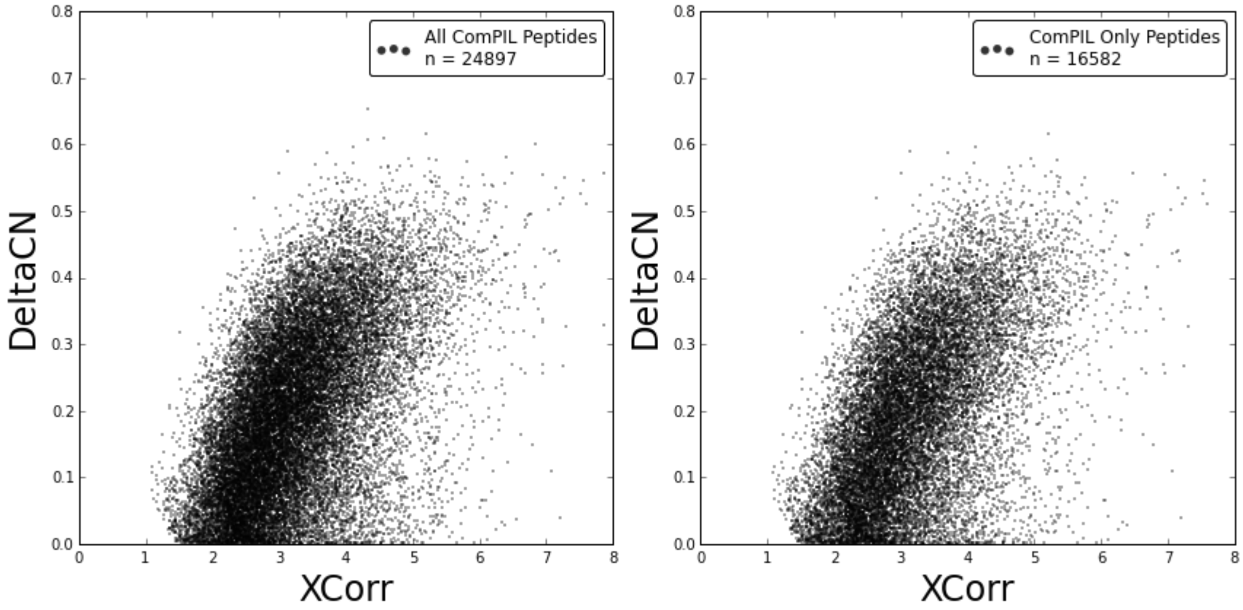

Figure S8 - DeltaCN vs. XCorr for the ComPIL vs. 46 proteomes search of the H1_1 fecal microbiome sample. All peptides found in the ComPIL search are shown (left), along with the peptides that are found in the ComPIL search only (not found in the 46 proteomes search) (right).

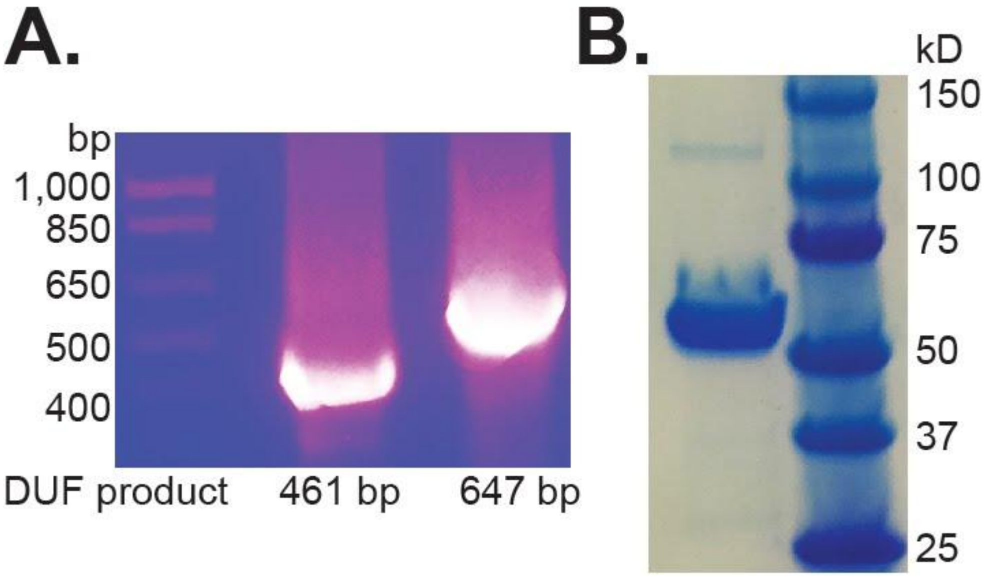

Figure S9 – Confirmation of *B. wexlerae* DUF PF09861. A) Representative amplification of *B. wexlerae* DUF gene segments from microbial DNA obtained from health sample #4 fecal sample. B) Purified exogenously expressed full length *B. wexlerae* DUF PF09861.

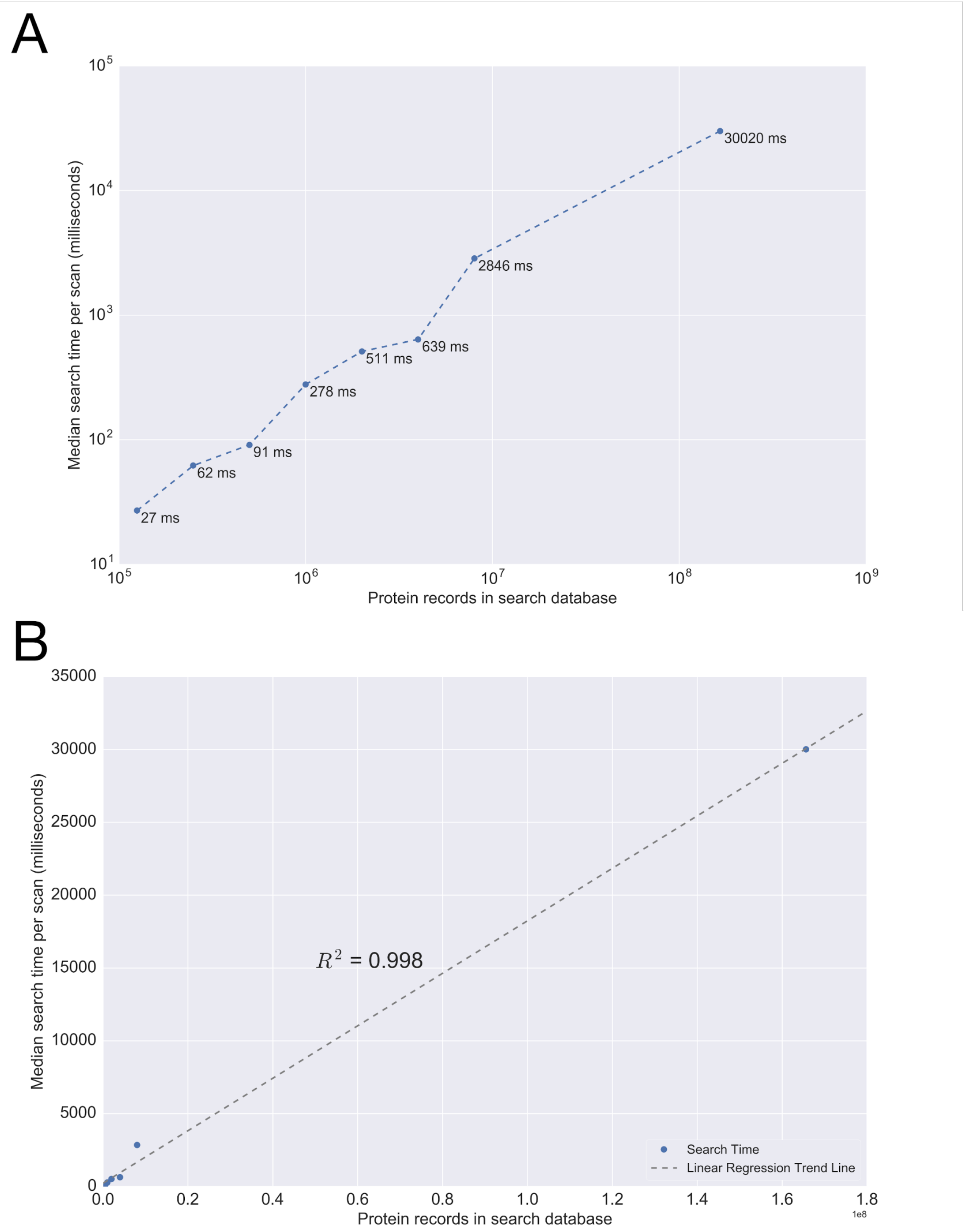

Figure S10 - Relationship between database size and search time for a Blazmass search using different sized ComPIL databases. The median search time per scan in milliseconds is plotted versus the number of proteins in the database (A) on a log-log scale and (B) on a linear scale The relationship is approximately linear within this range. For reference, the 46 proteomes database has 949,024 proteins and our proof-of-concept ComPIL database has 165 million proteins.

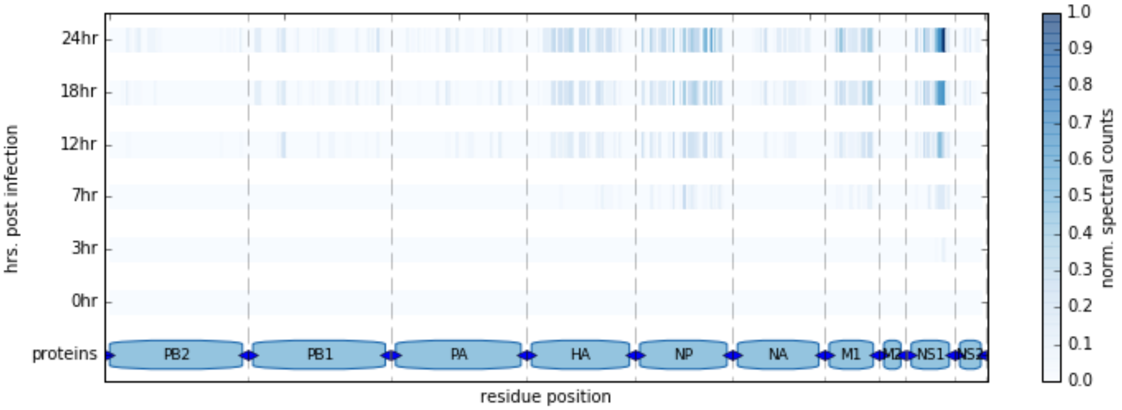

Figure S11 – Detection of Influenza A peptides in infected Calu-3 cells searched using the human and A/Anhui/1/2013 proteome. Detected Influenza peptides are shown mapped to their location within the Influenza A/Anhui/1/2013 proteome. The color represents the normalized spectral counts of peptides found at each residue. Peptides that corresponded to expression of neuraminidase and NS2, which were not seen observed with the ComPIL search are detected. This is most likely due to low sequence identity between the Anhui strain and other Influenza A strains in ComPIL.

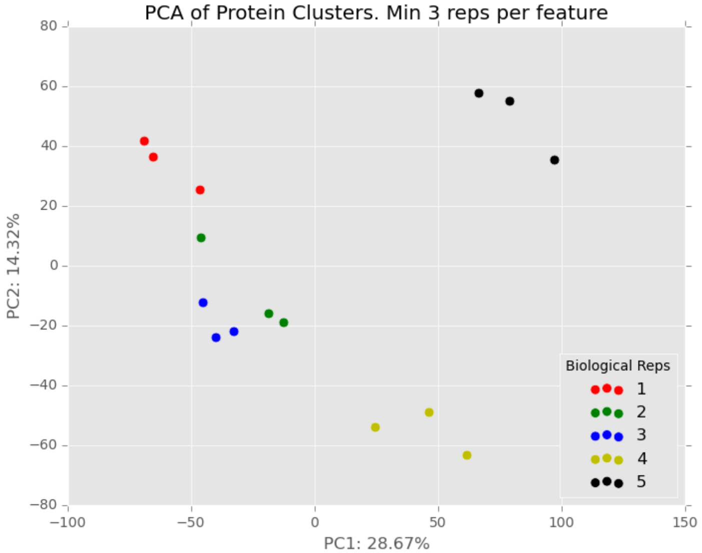

Figure S12 – Principal Component Analysis scores plot of 15 human gut microbiome samples. Shown are 3 technical replicates each of 5 biological replicates (each colored). Features are spectral counts of protein clusters.
